# Dynamic transmission and evolutionary analysis of the HIV-1 subtype CRF01_AE pol region in Ningxia, China

**DOI:** 10.1371/journal.pone.0354910

**Published:** 2026-07-29

**Authors:** Ziyang Luo, Wei Sun, Jianxin Pei, Subinuer Mutalifu, Youping Duan, Yufeng Li, Xiaohong Zhu, Zhonglan Wu

**Affiliations:** 1 College of Public Health, Ningxia Medical University, Yinchuan, China; 2 Ningxia Administration of Disease Control and Prevention, Yinchuan, China; 3 Ningxia Center for Disease Control and Prevention, Yinchuan, China; 4 College of Life Sciences, Ningxia University, Yinchuan, China; Centers for Disease Control and Prevention, UNITED STATES OF AMERICA

## Abstract

The CRF01_AE recombinant is the dominant HIV strain in Ningxia, characterized by rapid disease progression and imposing a significant local burden. Current prevention strategies, often based on broad subtype classifications, are insufficient to address the distinct transmission dynamics of such specific recombinants. To delineate the spatiotemporal migration patterns and epidemic trends of CRF01_AE in Ningxia, this study conducted a systematic analysis of 206 local sequences. The results reveal extensive viral spread with clear geographic diffusion routes, particularly strong migration from Yinchuan to other major cities. Key demographic groups driving transmission are individuals aged 20–39, retired/unemployed persons, and those with junior high school education. Although the transmission intensity has fluctuated, the current effective reproductive number remains above 1, indicating ongoing epidemic expansion, while the viral evolutionary rate appears stable. The study demonstrates that the CRF01_AE epidemic in Ningxia is still expanding and evolving, and identifies core transmission hubs and population clusters. These findings not only align with some prior understanding but also uncover more complex transmission networks and demographic patterns, highlighting the necessity for implementing targeted interventions specific to this strain to effectively control its spread.

## Introduction

Human immunodeficiency virus type 1 (HIV-1) remains a major global public health challenge, with cumulative infections reaching approximately 40.8 million people by the end of 2024 [1]. Among the circulating strains, subtype C is the most prevalent, accounting for 46.6% of cases, followed by subtypes B and A at 12.1% and 10.3%. Notably, recombinant forms—including circulating recombinant forms (CRFs) and unique recombinant forms (URFs)—collectively represent 22.8% of global infections, with CRF01_AE and CRF02_AG comprising 5.3% and 7.7%, respectively [2]. Many studies have extensively documented the global distribution of HIV-1 recombinants, highlighting CRF01_AE as the predominant strain in East and Southeast Asia [2,3]. Importantly, recombinant viruses are increasing in proportion at a significantly higher rate compared to pure subtypes in Asia, suggesting that they may possess enhanced transmissibility, adaptability, or replicative capacity [3].

Since 2020, CRF01_AE and CRF07_BC have emerged as the predominant HIV-1 strains in China. According to HIV testing data from the Ningxia Center for Disease Control and Prevention, the HIV epidemic in the Ningxia is characterized by the coexistence of multiple recombinant strains, including CRF07_BC, CRF01_AE, CRF08_BC, and others. CRF07_BC remains the most prevalent subtype, accounting for 53.2% of cases, followed by CRF01_AE at 25.5%. The majority of cases are concentrated in Yinchuan City, with heterosexual transmission being the predominant route. In the northwestern region where Ningxia is located, the prevalence of CRF07_BC has been gradually declining, while other subtypes are accounting for an increasing proportion of infections [4]. Among these, CRF01_AE has drawn particular attention due to its association with more rapid disease progression. Studies indicate that individuals infected with CRF01_AE experience the fastest decline in CD4^+^T cell counts to 200 cells/μl from the time of diagnosis and require a longer duration to achieve immune recovery [5,6]. These findings highlight the need for earlier identification and longer, more closely monitored treatment periods for patients with this subtype. Therefore, more precise, subtype-specific prevention and control strategies should be implemented for confirmed CRF01_AE cases to mitigate its aggressive clinical course.

In this study, we conducted Bayesian phylogenetic analysis and reproductive number estimation on the pol region sequences of all HIV-1 subtype CRF01_AE patients in Ningxia to determine viral migration patterns across different populations and evaluate the evolutionary tendency. This provides theoretical and data support for tracking the evolution of CRF01_AE and precision prevention and control of high risk populations.

## Methods

### Study subjects

Plasma samples were collected from HIV/AIDS patients in Ningxia between 2007 and 2024, including both those who had received antiretroviral therapy (ART) and those who were newly diagnosed and had not yet initiated ART. All samples were stored at −80°C. From these, samples with viral loads >400 copies/mL were selected for further analysis. RNA was extracted from the plasma using an automated nucleic acid extraction and purification system along with its matching reagent kit (Zhuhai Livzon Diagnostics Inc., Zhuhai, China) [7]. Using an in-house method [8,9], the full-length protease region and the first 300 amino acid codons of the reverse transcriptase gene in the HIV-1 pol region were amplified, yielding a fragment approximately 1,100 bp in length. The obtained sequences were compiled and submitted to the HIV database via the online HIV BLAST tool (https://blast.ncbi.nlm.nih.gov/Blast.cgi) for preliminary subtyping analysis. A maximum likelihood phylogenetic tree was constructed using MEGA12 software to confirm the subtype classifications. Finally, all the samples with the identification result of CRF01_AE were included, resulting in 206 sequences. These sequences were then transformed into temporal sequences, which were used as the research subjects of this study. Both the demographic information and sequence data for this research were accessed on 05/12/2025.

### Statistical analysis

Categorical data were reported as numbers and percentages, and group comparisons were conducted using SPSS 29.0 with Fisher’s exact test when more than 20% of the cells in the table had an expected count of less than 5, or when any cell had an expected count of less than 1.

### Evolutionary characteristics and phylogeographic inference

To characterize the transmission dynamics of CRF01_AE subtype across different age cohorts and geographic regions, we implemented an integrated Bayesian phylogenetic framework combining multiple computational approaches. First, we established the temporal sequences. Then, we performed the the sequence alignment using MEGA12 by maximum likelihood phylogenetic reconstruction to generate a Newick file. Next we imported the Newick file into TempEst v1.5.3 software to assess temporal signal by Best-fitting root, identify the problematic sequences through the extreme values in the scatter plot and residual plot, and correct or delete them, then requiring a correlation coeffcient greater than 0.3 [10].

### Bayesian phylogenetic analysis

We configured Skygrid model parameters under a general time-reversible (GTR) substitution model with an uncorrelated relaxed clock using BEAUti v10.5.0 to generate the maximum clade credibility (MCC) tree. Convergence was assessed by ensuring effective sample size (ESS) values ≥200, as determined in Tracer v1.7.2. The Bayesian stochastic search variable selection (BSSVS) program was employed to identify relationships between subgroups, while Markov jumps were used to calculate the expected number of viral migrations. Bayesian factors and posterior probabilities were computed using SpreaD3 v0.9.6, with subsequent analysis restricted to results meeting stringent criteria (Bayesian factors ≥3 and posterior probabilities ≥0.8).

### Systematic-dynamical analysis

We established Birth-Death Skyline model (BDSKY) [11] serial parameters under a GTR substitution model with a Relaxed Clock log Normal using BEAUti v2.7.7 to generate XML files, which were then executed in BEAST v2.7.7. After verifying ESS values ≥200 in Tracer v1.7.2, the log files were processed in R v4.4.0 using the “bdskytools” package to dynamically visualize Re values, reconstruct transmission dynamics and their 95% highest probability density (95%HPD) [12]. This approach provided temporal insights into the epidemic spread of subtype CRF01_AE.

To ensure the robustness of the evolutionary rate estimation, we first re‑identified DRM (Drug Resistance Mutation) codons in all sequences using the Stanford HIVdb database and masked those with a frequency >1% (S1 Table). Then, using the BDSKY model and after confirming in Tracer v1.7.2 that the ESS of all parameters were ≥200, we extracted the molecular clock‑related parameters to compute the evolutionary rate.

### Ethics and consent

The research protocol of the present study was approved by the Institutional Review Board of the Ningxia Hui Autonomous Region Center for Disease Control and Prevention (No. 2025‑LLSC‑228).

## Results

### Comparison of demographic

This study collected data on 206 CRF01_AE patients reported in Ningxia by the end of 2024. The major characteristics of them, males (88.9%), from Yinchuan District (57.3%), aged 20–39 (43.7%), heterosexual transmission (68.5%), had junior high school education (31.2%), and were farmers (30.1%). Results showed statistically significant differences in the route of infection and drug resistance across different diagnosis time periods(Table 1). Only those cases with more than two types of features were included in the subsequent construction of the virus migration model.

**Table 1:** Demographic characteristics and temporal distribution of CRF01_AE subtype cases in Ningxia.

	Total(%)	Diagnosis time				*p* values
		<2012	2012-2016	2016-2020	2020-2024	
Gender						0.806^a^
male	183(88.9)	4(80.0)	27(90.0)	57(89.1)	95(88.8)	
female	23(11.1)	1(20.0)	3(10.0)	7(10.9)	12(11.2)	
Age						0.278^a^
<20	5(2.4)	1(20.0)	1(3.3)	0(0.0)	3(2.8)	
20 ~ 39	90(43.7)	1(20.0)	17(56.7)	29(45.3)	43(40.2)	
40 ~ 59	80(38.8)	3(60.0)	9(30.0)	24(37.5)	44(41.1)	
≥60	31(15.1)	0(0.0)	3(10.0)	11(17.2)	17(15.9)	
Occupation						0.067^a^
Unemployed/retiree	62(30.1)	0(0.0)	10(33.3)	14(21.9)	38(35.6)	
Farmer	62(30.1)	0(0.0)	8(26.7)	22(34.4)	32(29.9)	
Business waiter	24(11.7)	1(20.0)	2(6.7)	12(18.7)	9(8.4)	
Workers	22(10.7)	1(20.0)	6(20.0)	5(7.8)	10(9.3)	
Others and unknown	36(17.4)	3(60.0)	4(13.3)	11(17.2)	18(16.8)	
Route of infection						0.035^a^
Heterosexual	141(68.5)	2(40.0)	16(53.4)	49(76.6)	74(69.2)	
Homosexual	60(29.1)	2(40.0)	13(43.3)	13(20.3)	32(29.9)	
others	5(2.4)	1(20.0)	1(3.3)	2(3.1)	1(0.9)	
Degree of education						0.120^a^
Illiterate	20(9.7)	1(20.0)	2(6.8)	6(9.3)	11(10.3)	
Primary school	32(15.5)	0(0.0)	4(13.3)	14(21.9)	14(13.1)	
Junior high school	64(31.2)	1(20.0)	10(33.3)	25(39.1)	28(26.2)	
High school	45(21.8)	0(0.0)	10(33.3)	8(12.5)	27(25.2)	
College degree	45(21.8)	3(60.0)	4(13.3)	11(17.2)	27(25.2)	
Drug resistance						0.007^a^
Yes	77(37.4)	4(80.0)	16(53.4)	27(42.2)	30(28.0)	
No	129(62.6)	1(20.0)	14(46.6)	37(57.8)	77(72.0)	
Place of residence						0.802^a^
Yinchuan City	118(57.3)	3(60.0)	16(53.4)	36(56.2)	63(58.9)	
Shizuishan City	22(10.7)	1(20.0)	4(13.3)	7(10.9)	10(9.3)	
Wuzhong City	11(5.3)	0(0.0)	1(3.3)	1(1.6)	9(8.4)	
Guyuan City	25(12.1)	1(20.0)	4(13.3)	9(14.1)	11(10.3)	
Zhongwei City	30(14.6)	0(0.0)	5(16.7)	11(17.2)	14(13.1)	

Note: *p*^a^ values were obtained from Fisher test.

### Viral migration patterns

**Fig 1:**
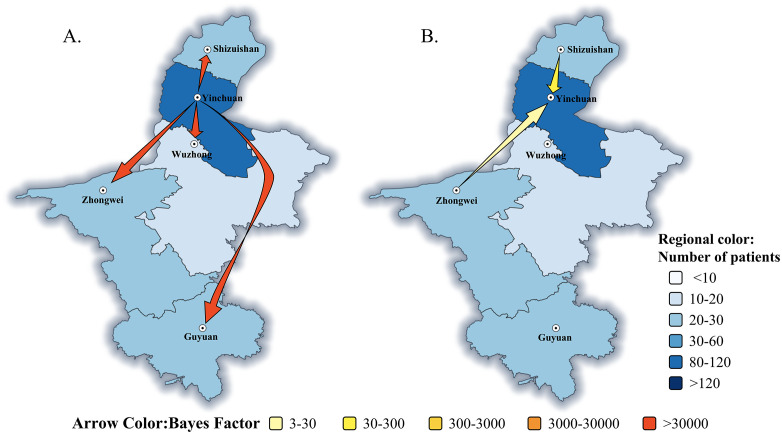
The geographical migration patterns of the HIV-1 CRF01_AE in Ningxia, China. The different regions are colored according the number of patients with HIV-1 subtype CRF01_AE (Note: The base map is from the China National Bureau of Surveying, Mapping and Geoinformation which provides a standard map download service, Review No. GS(2024)0650).

Based on Bayesian phylogenetic analysis, this study reconstructs the historical transmission patterns of the CRF01_AE virus across different populations and geographic regions. Geographically, strong viral migration events (Bayes Factor, BF > 30,000) were predominantly centered in Yinchuan City, with subsequent spread to the four other cities — Shizuishan, Wuzhong, Guyuan, and Zhongwei(Fig 1A). In addition, weak migration signals (BF < 300) from Zhongwei and Shizuishan back to Yinchuan were identified, suggesting a limited degree of viral backflow into the initial epicenter(Fig 1B).

**Fig 2:**
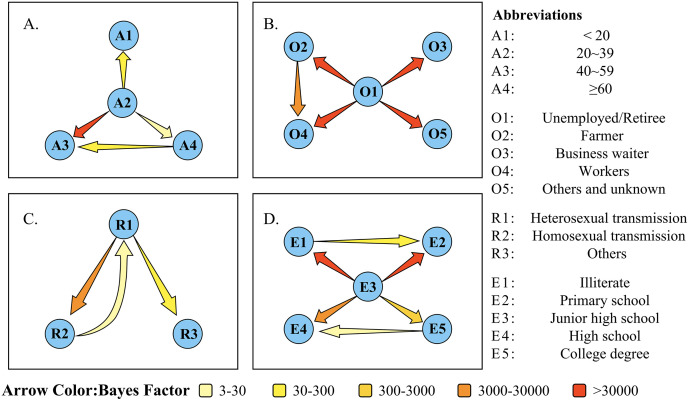
The migration events of HIV-1 subtype CRF01_AE. Presented HIV migration events among different characteristic groups in Age(A), Occupation(B), Route of infection(C), Degree of education(D). We only report well-supported HIV-1 migration events, defined by a Bayes factor (BF) ≥ 3 and a posterior probability ≥ 0.8. Arrows indicate migration direction, and colors represent different levels of BF support.

From the perspective of population characteristics, CRF01_AE exhibits a migration pattern primarily centered in the 20–39 age group, with outward spread to the < 20, 40–59, and ≥60 age groups. A weaker reverse migration is also observed from the ≥ 60 group back to the 40–59 age group (Fig 2A). In terms of occupational classification, the main viral migration originates from the unemployed or retired group to the other four categories: farmers, business service waiter, general workers, and others. A secondary transmission pathway was also identified from farmers to general workers (Fig 2B). Regarding transmission routes, bidirectional migration is evident between the homosexual and heterosexual transmission populations. The heterosexual group also serves as a source of spread to other transmission categories (Fig 2C). Analysis of educational attainment reveals a complex network of viral migration. Key pathways include movement from junior high school to illiteracy, primary school, high school, and college. This network is further complicated by cross-level flows, such as from illiteracy group to the primary school-educated group and from college-educated group back to the high school-educated group (Fig 2D).

### Modelling effective reproductive number

Phylogenetic analysis reveals that when modeling at the regional level of Ningxia, the evolutionary rate of the CRF01_AE subtype is estimated at 3.006 × 10^-3^ subs./site/year (95%HPD: 2.370 × 10^-3^-3.686 × 10^-3^ subs./site/year), with a mean reproductive number (Re) of 1.340 (95%HPD: 1.285–1.393). When narrowing the scope to individual prefecture-level cities, the following estimates are observed: in Yinchuan City, the evolutionary rate is 2.556 × 10^-3^ subs./site/year (95%HPD: 1.866 × 10^-3^-3.397 × 10^-3^ subs./site/year), while the Re remains 1.340 (95%HPD: 1.270–1.412); in Shizuishan City, the evolutionary rate increases to 6.852 × 10^-3^ subs./site/year (95%HPD: 5.129 × 10^-3^-8.721 × 10^-3^ subs./site/year), with an Re of 1.074 (95%HPD: 0.757–1.404); Wuzhong City exhibits an evolutionary rate of 6.439 × 10^-3^ subs./site/year (95%HPD: 4.346 × 10^-3^-8.620 × 10^-3^ subs./site/year) and an Re of 1.139 (95%HPD: 0.672–1.654); Guyuan City shows a lower evolutionary rate of 4.913 × 10^-3^ subs./site/year (95%HPD: 3.688 × 10^-3^-6.319 × 10^-3^ subs./site/year), accompanied by an Re of 1.049 (95%HPD: 0.774–1.303); and Zhongwei City has an evolutionary rate of 3.331 × 10^-3^ subs./site/year (95%HPD: 2.402 × 10^-3^-4.299 × 10^-3^ subs./site/year) and an Re of 1.196 (95%HPD: 0.943–1.446). Among all models, the one based on Ningxia as a unit yielded the highest Re value, and although its evolutionary rate was not the highest, it was the most stable, with a narrow HPD range indicating high reliability of the results (Table 2).

**Table 2:** Re and evolationary rate.

	Re	95%HPD	Evolutionary Rate (subs./site/year)	95%HPD (subs./site/year)
Ningxia	1.340	1.285-1.393	3.006 × 10^−3^	2.370 × 10^-3^-3.686 × 10^−3^
Yinchuan City	1.340	1.270-1.412	2.556 × 10^−3^	1.866 × 10^-3^-3.397 × 10^−3^
Shizuishan City	1.074	0.757-1.404	6.852 × 10^−3^	5.129 × 10^-3^-8.721 × 10^−3^
Wuzhong City	1.139	0.672-1.654	6.439 × 10^−3^	4.346 × 10^-3^-8.620 × 10^−3^
Guyuan City	1.049	0.774-1.303	4.913 × 10^−3^	3.688 × 10^-3^-6.319 × 10^−3^
Zhongwei City	1.196	0.943-1.446	3.331 × 10^−3^	2.402 × 10^-3^-4.299 × 10^−3^

**Fig 3:**
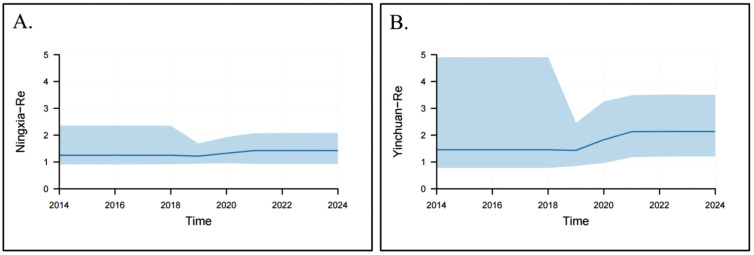
Trend Analysis of reproductive number (Re) and their 95%HPD.

Regardless of the modeling parameters applied, the Re trend analysis for the CRF01_AE subtype pinpointed 2018–2020 as a consistent epidemiological shift, indicating a definitive turning point in its transmission dynamics. When Ningxia is considered as a whole, the effective reproduction number (Re) consistently exceeds 1, with a minor upward inflection observed around 2019(Fig 3A). A similar turning point occurred in Yinchuan City after downscaling the modeling unit, where Re also began to rise in 2019(Fig 3B).

## Discussion

This study delineates the virus migration patterns and evolutionary trajectories within the CRF01_AE subtype in Ningxia, China, which is of great significance for understanding the transmission characteristics of single CRF in the Ningxia region and for epidemic control. Compared with previous studies on all HIV-1 subtypes, the key populations identified in the CRF01_AE migration networks were more diverse. Meanwhile, based on the Re and evolution rate of CRF01_AE, the disease control department can optimize intervention measures and formulate more targeted prevention and control measures for CRF01_AE.

This study is the first to utilize the bioinformatics of HIV-1 to construct a model in Ningxia, revealing the migration patterns and trends of the CRF01_AE subtype of HIV-1. From a geographical perspective, the migration pattern has seen a strong shift from Yinchuan to the surrounding cities. This precisely conforms to the geographical transmission characteristics of infectious diseases—hierarchical spatial spread [13]. Infectious diseases tend to first emerge in densely populated large cities, and then spread along transportation routes to surrounding medium-sized cities and rural areas [14]. Yinchuan City, as the transportation and economic center of Ningxia, is spreading the CRF01_AE as a virus migration center to surrounding cities. Meanwhile, Shizuishan city and Zhongwei city are also conducting reverse migration towards Yinchuan city, which has led to the continuous expansion of the CRF01_AE virus in these two cycles. This phenomenon of reverse viral migration from peripheral cities back to the regional center might be related to rapid urbanization [15].

From the perspective of demographic characteristics, the migration pattern of CRF01_AE is also consistent with the previous research results on key populations of HIV-1. The migration pattern indicates that the age group has shifted from 20−39 years old to other age groups, and the occupational group has shifted from the unemployed and retired to other occupations. Our findings also encompass groups that are current research hotspots in HIV-1, namely college students and the elderly [16]. In many regions and countries, the incidence of new infections among teenagers and young adults has shown a worrying increase. This is mainly due to the inadequate coverage of comprehensive sex education and the sexual urges of young people [17]. As the widespread use of ART for elderly AIDS patients has led to an increase in survival rates, the size of this group has grown. At the same time, the sexual behaviors of the elderly have been overlooked, resulting in the gradual expansion of the virus migration pattern starting from the elderly [18]. Our findings provides strong support for the mentioned high risk groups while also covering a broader population, and offers more appropriate recommendations for the prevention and control of CRF01_AE in the general population.

In previous studies, the virus migration pattern of CRF01_AE started from the men who have sex with men(MSM) population and then spread to drug users or intravenous drug users, and eventually reached their partners [19]. However, due to the shame associated with homosexuality in China, some MSM individuals conceal their true sexual orientation, leading to the phenomenon of “marriage fraud” [20]. Among the heterosexual transmission population, there are many potential men who have sex with men(PnMSM) population. The phenomenon of heterosexual transmission population migrating to the MSM population is essentially internal transmission within the MSM population, except that some MSM have hidden their identities [21]. As a result, heterosexual transmission occurs in the middle of the migration chain rather than at the end. This phenomenon not only constitutes deception towards partners but also poses significant risks to them [22]. Moreover, it introduces uncertainty in identifying key populations for HIV-1 prevention and control. Although MSM are internationally recognized as high risk groups, in China, the scope of identification still needs to be expanded.

The virus migration patterns related to educational attainment shift from the junior high school education level to other categories, but the strong migration（BF > 30000) only occurs below the junior high school education level. A study conducted in Chicago, USA, indicates that in areas with a stronger cultural background and community structure, the likelihood of having HIV-1positive individuals is lower [23]. High-quality communities and cultural environments typically signify better educational levels, so the migration of the HIV-1 virus among those with low educational attainment is often more pronounced.

The model of this study indicates that the overall reproduction number in Ningxia region is still greater than 1 at present, which proves that the CRF01_AE virus is still spreading continuously. Since the introduction of CRF01_AE from Southeast Asia in the 1990s [24], it has evolved over several decades into seven major phylogenetic clusters [25]. One of the most significant phenotypic research findings is that CRF01_AE has a faster disease progression and causes a more rapid decline in CD4^+^T cells compared to other subtypes [26,27]. Meanwhile, domestic studies have shown that the second-generation recombinant (SGR) based on CRF01_AE has emerged, and three similar new HIV SGR strains have been found in Shenzhen, China [28]. When focusing the study scope on Ningxia, the effective reproduction numbers of multiple subtypes show sustained growth, indicating that HIV remains not yet fully under control in the Ningxia region [29]. All these studies highlight the importance of detecting the genetic evolution and phenotypic changes of CRF01_AE and has raised concerns that CRF01_AE may evolve into a more pathogenic strain during its continuous spread. However, from an evolutionary perspective, although the pol region of CRF01_AE is still evolving, the rate of evolution remains at a level of ten to the third, which is within the normal range or even slower, consistent with other domestic studies [30], this proves that the evolution of CRF01_AE remains stable.

## Conclusions

According to the model, the HIV-1 CRF01_AE subtype in the Ningxia region is still on the rise, but its evolutionary rate at the genetic level remains stable. Research and prevention efforts for CRF01_AE should not be limited to college students, the elderly, and MSM. Instead, it has further expanded the distinction of previous prevention target groups. This will help to formulate unique prevention strategies for different CRFs in the subsequent HIV prevention policies, rather than blindly assuming that the key prevention directions for all subtypes are the same, especially in an era when the proportion of CRFs is gradually increasing.

## Supporting information

S1 Table Frequency of drug resistance mutations in CRF01_AE patients in Ningxia region. (DOCX)

## Abbrevations:

HIV-1: Human immunodeficiency virus type 1
CRFs: Circulating recombinant forms
URFs: Unique recombinant forms
ART: Antiretroviral therapy
MCC: Maximum clade credibility
ESS: Effective sample size
BSSVS: Bayesian stochastic search variable selection
BF: Bayes Factor
BDSKY: Birth-Death Skyline model
95%HPD: 95% highest probability density
Re: Effective reproduction number
MCMC: Markov chain monte carlo
MSM: Men who have sex with men
PnMSM: Potential men who have sex with men
SGR: Second-generation recombinant
DRM: Drug Resistance Mutation
