# Supplementary material for: Dynamic transmission and evolutionary analysis of the HIV-1 subtype CRF01_AE pol region in Ningxia, China
Source: PLoS One. 2026 Jul 29;21(7):e0354910. doi: 10.1371/journal.pone.0354910 (PMC13419192; doi:10.1371/journal.pone.0354910)
Supplement: S1 Table — (DOCX) [file pone.0354910.s001.docx]

Supplementary tables

S1 Table. Frequency of drug resistance mutations in CRF01_AE patients in Ningxia region

| Drug resistance mutations | | Total (column %) | Drug resistance mutations | | Total (column %) |
| --- | --- | --- | --- | --- | --- |
| PI | T74TP | 2(0.68%) | NNRTI | K103N | 21(7.12%) |
|  | L33F | 1(0.34%) |  | V106M | 13(4.41%) |
|  | L89IMT | 1(0.34%) |  | V179D | 13(4.41%) |
| NRTI | M184V | 22(7.46%) |  | Y181C | 11(3.73%) |
|  | K65R | 16(5.42%) |  | G190S | 11(3.73%) |
|  | S68G | 16(5.42%) |  | K101E | 10(3.39%) |
|  | M184I | 7(2.37%) |  | V179T | 10(3.39%) |
|  | D67N | 7(2.37%) |  | A98G | 4(1.36%) |
|  | Y115F | 6(2.03%) |  | G190A | 3(1.02%) |
|  | A62V | 5(1.69%) |  | K101EQ | 3(1.02%) |
|  | K70E | 5(1.69%) |  | K238T | 3(1.02%) |
|  | M184MI | 5(1.69%) |  | L100I | 3(1.02%) |
|  | K65KR | 4(1.36%) |  | M230L | 3(1.02%) |
|  | S68SG | 4(1.36%) |  | P225H | 3(1.02%) |
|  | T215TS | 4(1.36%) |  | Y181YC | 3(1.02%) |
|  | A62AV | 3(1.02%) |  | F227L | 2(0.68%) |
|  | D67DN | 3(1.02%) |  | G190GS | 2(0.68%) |
|  | K219Q | 3(1.02%) |  | H221Y | 2(0.68%) |
|  | M184MV | 3(1.02%) |  | K103KN | 2(0.68%) |
|  | S68N | 3(1.02%) |  | V106I | 2(0.68%) |
|  | Y115YF | 3(1.02%) |  | V106VI | 2(0.68%) |
|  | K70Q | 2(0.68%) |  | V108I | 2(0.68%) |
|  | K219R | 2(0.68%) |  | V108VI | 2(0.68%) |
|  | L74LI | 2(0.68%) |  | Y188L | 2(0.68%) |
|  | M41L | 2(0.68%) |  | E138A | 1(0.34%) |
|  | D67G | 1(0.34%) |  | G190C | 1(0.34%) |
|  | E44D | 1(0.34%) |  | H221HY | 1(0.34%) |
|  | K65N | 1(0.34%) |  | K101KE | 1(0.34%) |
|  | K70G | 1(0.34%) |  | K101P | 1(0.34%) |
|  | K70KE | 1(0.34%) |  | K103Q | 1(0.34%) |
|  | K70KT | 1(0.34%) |  | K238KNRS | 1(0.34%) |
|  | K70N | 1(0.34%) |  | P225PH | 1(0.34%) |
|  | K70R | 1(0.34%) |  | V106A | 1(0.34%) |
|  | K70T | 1(0.34%) |  | V179DE | 1(0.34%) |
|  | K219E | 1(0.34%) |  | V179E | 1(0.34%) |
|  | K219N | 1(0.34%) |  | V179VD | 1(0.34%) |
|  | L74I | 1(0.34%) |  | V179VF | 1(0.34%) |
|  | L74LV | 1(0.34%) |  | Y181I | 1(0.34%) |
|  | M184MIV | 1(0.34%) |  |  |  |
|  | S68SDGN | 1(0.34%) |  |  |  |
|  | S68SN | 1(0.34%) |  |  |  |
|  | T215FL | 1(0.34%) |  |  |  |
|  | T215Y | 1(0.34%) |  |  |  |
